# Association of HBV DNA replication with antiviral treatment outcomes in the patients with early-stage HBV-related hepatocellular carcinoma undergoing curative resection

**DOI:** 10.1186/s40880-016-0089-z

**Published:** 2016-03-18

**Authors:** Jian-Lin Chen, Xiao-Jun Lin, Qian Zhou, Ming Shi, Sheng-Ping Li, Xiang-Ming Lao

**Affiliations:** Department of Hepatobiliary Oncology, State Key Laboratory of Southern China, Collaborative Innovation Center for Cancer Medicine, Sun Yat-sen University Cancer Center, 651 Dongfeng Road East, Guangzhou, 510060 Guangdong P. R. China; Epidemiology Research Unit, The First Affiliated Hospital of Sun Yat-sen University, Guangzhou, 510080 Guangdong P. R. China

**Keywords:** Hepatocellular carcinoma, Resection, Hepatitis B virus, Prognosis, Antiviral therapy

## Abstract

**Background:**

It remains unclear what the antiviral therapy affects disease-free survival (DFS) and overall survival (OS) of patients with hepatitis B virus (HBV)-related hepatocellular carcinoma (HCC) at different tumor stages and baseline HBV DNA levels. In this study, we analyzed the association of antiviral treatment with DFS and OS based on the stratification of baseline HBV DNA load in early-stage (stages I and II) HCC patients.

**Methods:**

We included 445 patients with early-stage HBV-related HCC who underwent curative resection, and then classified them into four subgroups based on baseline HBV DNA load and antiviral therapy stratification. The Kaplan–Meier and Cox regression analyses were performed to determine the association of clinical characteristics with survival.

**Results:**

The median follow-up period was 74 months. For all patients, cumulative OS rates in the antiviral group were significantly higher than those in the non-antiviral group (log-rank test, *P* = 0.023), whereas no significant differences in DFS rates were observed. High baseline HBV DNA level was a risk factor associated with short DFS and OS in all patients. In patients with baseline HBV DNA levels ≥2000 IU/mL, antiviral treatment was significantly associated with prolonged DFS and OS (log-rank test, *P* = 0.041 and 0.001, respectively). In patients with HBV DNA levels <2000 IU/mL or undetectable, antiviral treatment did not show a significant benefit in prolonging DFS and OS.

**Conclusions:**

High baseline HBV DNA levels are associated with poor prognosis in the patients with early-stage HCC, and the antiviral treatment could generate survival benefits for the patients. Therefore, antiviral treatment should be given for these patients. However, the effect of antiviral treatment on the patients with low viral load remains unclear, and further investigation is warranted.

## Background

Hepatocellular carcinoma (HCC) is the fifth most common cancer in men and the seventh most common cancer in women, and it is the third most common cause of cancer-related death worldwide [1]. The 2010 incidence and mortality of liver cancer in China were among high levels worldwide [2]. Most HCCs develop within an established background of chronic liver disease, and the most common risk factor for HCC is hepatitis B virus (HBV) infection [3]. High levels of serum HBV DNA are associated with an increased risk of developing HCC [4], and nomograms based on clinical characteristics, including serum hepatitis B e antigen (HBeAg) status and HBV DNA level, can predict the risk of developing HCC [5]. Additionally, in HBV-related HCC, high levels of serum HBV DNA appear to be associated with poor prognosis [6–8]. Therefore, careful management of HBV infection is needed in the management of HBV-related HCC [9].

Hepatic resection has been the standard curative treatment for HCC. However, a high rate of tumor recurrence has been reported after surgery [10]. So far, no effective adjuvant treatment option has been proven to reduce the risk of recurrence [11]. Recently, some studies demonstrated that antiviral therapy with nucleotide/nucleoside analogs (NAs) was associated with prolonged disease-free survival (DFS) and overall survival (OS) after hepatectomy of HBV-related HCC, suggesting that antiviral treatment may offer clinical benefits in HCC patients with HBV infection [12–15]. However, most of these studies did not stratify the patients into specific subgroups based on tumor stages or HBV DNA levels, and the results of these studies were contradictory. Early-stage tumors tend to be associated with a better prognosis than intermediate-stage tumors, and antiviral treatment may confer a significant survival benefit compared with non-antiviral treatment [16, 17]. Another issue that should be considered is the HBV DNA load. Whether it is necessary to give antiviral treatment to HCC patients with low or even undetectable HBV DNA levels remains controversial [18, 19]. So far, no consensus on this issue exists, and the indications for antiviral treatment in patients with HBV-related HCC remain uncertain.

In this retrospective study, we used strict classification of patients into subgroups, comparison of DFS and OS, and multivariate analysis to (1) evaluate the effect of HBV DNA on the prognosis of patients with early-stage [stages I and II; Union for International Cancer Control (UICC), 7th edition] HCC who underwent curative therapy and (2) analyze the effect of antiviral treatment on DFS and OS based on the stratification of baseline HBV DNA load.

## Patients and methods

### Patients

This retrospective cohort study in a tertiary academic hospital was approved by the Institutional Review Board of Sun Yat-sen University Cancer Center in Guangdong, China. We included patients who were initially diagnosed with hepatitis B surface antigen (HBsAg)-positive HCC and underwent curative resection between January 2005 and December 2008 at the Hepatobiliary Department of Sun Yat-sen University Cancer Center. Prior to obtaining tissues, written informed consent was obtained from each patient.

### Preoperative evaluation

As described previously [20], baseline examinations (within 10 days before surgery), including serum HBV DNA quantification, detection of HBsAg, and liver function tests, were routinely performed. Serum HBV viral loads were measured by using a quantitative fluorescence polymerase chain reaction detection kit (DaAn Gene Corporation, Guangzhou, China) with a lower detection limit of 200 IU/mL. Dynamic contrast-enhanced computed tomography (CT) and/or magnetic resonance imaging (MRI) were used to assess the intrahepatic tumor. The preoperative diagnosis of HCC was based on the criteria established by the European Association for the study of the liver [21].

### Inclusion criteria

To be included in our study, patients had to meet the following criteria: (1) ≥18 years old, (2) hepatic resection for initial HCC treatment, (3) UICC TNM stage T1N0M0 or T2N0M0, (4) curative treatment (pathologic confirmation of negative resection margin) and no local recurrence within 3 months after surgery, (5) HBsAg positivity, (6) no anti-HBV treatment before surgery, (7) adequate baseline liver function (Child-Pugh grade A) and adequate renal function (serum creatinine <124 µmol/L), and (8) no tumor rupture before or during surgery.

### Exclusion criteria

Patients who met any of the following criteria were excluded: (1) HBsAg negativity, (2) evidence of co-infection with other hepatotropic viruses or human immunodeficiency virus, (3) any prior treatment for HCC, (4) Child-Pugh grade B or C, (5) presence of other malignancy or concurrent non-malignant severe illness, (6) tumor stage beyond T2N0M0, (7) recurrence or metastasis within 3 months after surgery, (8) interferon administration after surgery, (9) initiation of NA administration after tumor recurrence, and (10) severe complications or adverse events (including postoperative mortality) within 3 months after surgery.

### Tumor characteristics

The diagnosis of HCC was pathologically confirmed after surgery, and the histological differentiation of the tumors were graded from I to IV based on the Edmondson–Steiner classification [22]. Tumor number, maximal tumor size, tumor differentiation, tumor capsulation, the presence of microvascular invasion, and surgical margin were evaluated. Tumor characteristics and UICC TNM stage were comprehensively evaluated.

### Definition of curative resection

None of the cases had major vascular invasion. Curative resection was defined as complete removal of all macroscopically evident tumors [15, 23]. The absence of tumor cells along the parenchymal transection line was confirmed histologically. The CT or MRI examination performed 3 months after surgery showed no remaining tumor.

### Follow-up protocol

After discharge, each patient received regular examinations and follow-up at 3-month intervals until 3 years after resection, and at 4- to 6-month intervals thereafter. Follow-up ended on December 31, 2014. Serum liver function tests, alpha-fetoprotein (AFP) tests, chest X-ray radiography, abdominal ultrasonography, and CT/MRI scans were the routine examinations to determine possible recurrence or metastasis. Hepatic angiography, ultrasonic contrast, positron emission tomography/computed tomography, and ultrasound-guided biopsy were performed when necessary.

### Statistical analysis

Demographic data [mean, standard deviation, median (P25, P75), and percentage] were calculated. Analyses were conducted using the independent Student’s *t* test (Mann–Whitney test for non-normal distributions), analysis of variance (Kruskal–Wallis test for non-normal distributions), and the Chi square test, as appropriate. The Kaplan–Meier method and the log-rank test were used for survival analysis. DFS was the time calculated from the date of curative surgery to HCC recurrence, death, or the last follow-up; OS was the time calculated from the date of surgery to death or the last follow-up. The Cox proportional hazards model was used in the univariate survival analysis to determine the association of individual clinical variables with OS or DFS. All variables with *P* < 0.1 were subsequently subjected to the multivariate Cox regression model to determine the hazard ratios (HRs) and the independence of effects. *P* < 0.05 were considered statistically significant. All statistical tests were two-sided. Data were analyzed using the SAS 9.1 software (SAS Institute, Cary, NC, USA).

## Results

### General description

**Fig. 1:**
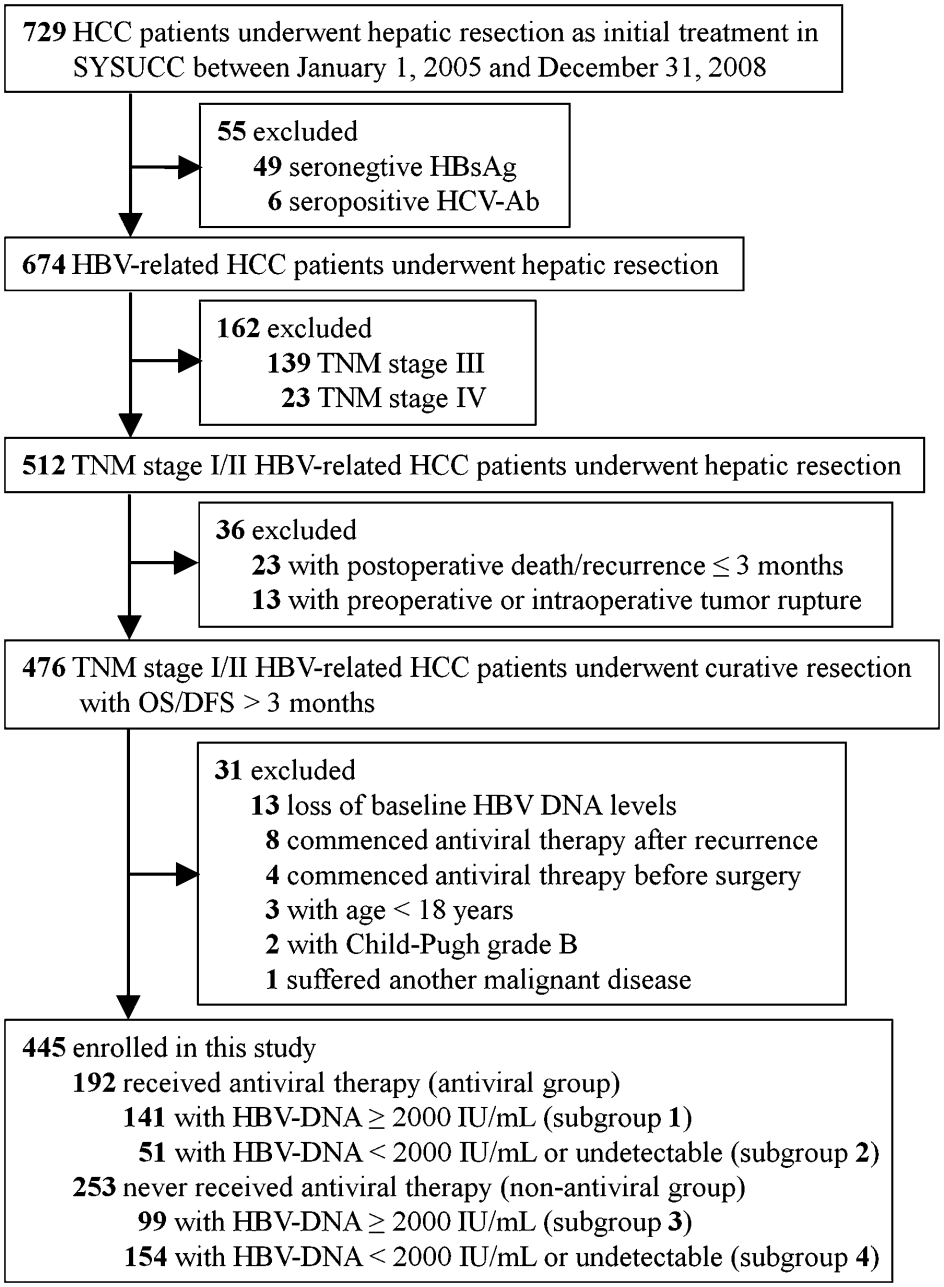
Selection of patients with early stage HBV-related HCC undergoing curative resection. *HCC* hepatocellular carcinoma, *SYSUCC* Sun Yat-sen University Cancer Center, *HBsAg* hepatitis B surface antigen, *HCV-Ab* hepatitis C virus antibody, *TNM* tumor-nodes-metastasis, *OS* overall survival time, *DFS* disease-free survival time, *HBV* hepatitis B virus

During the study period, 729 HCC patients underwent hepatic resection as their initial treatment at Sun Yat-sen University Cancer Center. According to our inclusion and exclusion criteria, 284 patients were excluded, and the remaining 445 patients were enrolled. The patient flow diagram is shown in Fig. 1.

In total, 192 patients received oral administration of NAs after surgery, with a median antiviral time of 79 months (P25, 47 months; P75, 87 months). Of these, 93, 84, 13, and 2 patients took entecavir, lamivudine, adefovir dipivoxil, and telbivudine as their initial antiviral agent, respectively. During the follow-up, 13 patients received sequential treatments due to partial HBV response or HBV resistance. The common treatment regimens involved lamivudine followed by entecavir-switch (four patients) and adefovir add-on (six patients). Other regimens included adefovir followed by entecavir-switch (two patients) and entecavir add-on (one patient).

Based on the stratification of baseline HBV DNA and antiviral therapy, the patients were classified into four subgroups: (1) subgroup 1, antiviral therapy with baseline HBV DNA ≥2000 IU/mL (*n* = 141); (2) subgroup 2, antiviral therapy with baseline HBV DNA <2000 IU/mL or undetectable (*n* = 51); (3) subgroup 3, no antiviral therapy with baseline HBV DNA ≥2000 IU/mL (*n* = 99); and (4) subgroup 4, no antiviral therapy with baseline HBV DNA <2000 IU/mL or undetectable (*n* = 154).

**Fig. 2:**
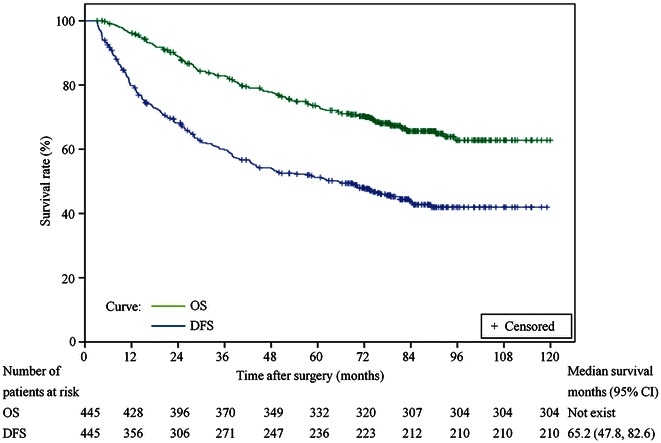
Kaplan–Meier survival curves for overall survival (OS) and disease-free survival (DFS) in patients who underwent curative resection (*n* = 445). *CI* confidence interval

Table 1 summarizes the characteristics of the antiviral group and non-antiviral group. Most variables were similar between these two groups. However, there were significant differences in HBeAg, HBV DNA, alanine aminotransferase (ALT), and serum total bilirubin levels, which may be explained by the fact that, in this retrospective study, patients with higher HBV replication or more severe liver damage tended to be treated with antiviral therapy. The median follow-up period was 74 months. Of the 445 patients, 235 (52.8 %) experienced tumor recurrence, and 141 (31.7 %) died during the follow-up period. The 1-, 3-, 5-, 7-, and 10-year OS rates in all study patients were 96.1 %, 82.6 %, 73.4 %, 65.6 %, and 62.8 %, respectively, whereas DFS rates were 79.8 %, 59.8 %, 51.2 %, 43.3 %, and 42.2 %, respectively (Fig. 2).

**Table 1:** Comparison of clinicopathologic characteristics between antiviral and non-antiviral groups

Characteristic	Antiviral group (*n* = 192)	Non-antiviral group (*n* = 253)	*P* value
Age (years)			0.372^a^
Mean ± SD^b^	47.9 ± 10.3	48.8 ± 11.8	
Median and range^b^	47 (24–76)	49 (23–79)	
<50	111	128	
≥50	81	125	
Gender			0.278
Male	172	218	
Female	20	35	
HBeAg			0.003
Positive	48	35	
Negative	144	218	
HBV DNA (IU/mL)			<0.001
<2000	51	154	
≥2000	141	99	
Tumor size (cm)			0.012^a^
Mean ± SD^b^	4.7 ± 3.1	5.4 ± 3.2	
Range^b^	1.0–25.0	1.0–18.0	
<5	122	121	
≥5	70	132	
Tumor number			0.990
Single	176	232	
Multiple	16	21	
Pathologic grade			0.961
I	12	21	
II	121	150	
III	58	80	
IV	1	2	
Microvascular thrombus			0.054
Yes	25	19	
No	167	234	
Tumor capsule			0.271
Complete	87	112	
Incomplete	60	66	
Without	45	75	
TNM stage			0.083
I	151	215	
II	41	38	
AFP (ng/mL)			0.881
≤25	78	101	
>25	114	152	
ALT (IU/L)			0.002^a^
Mean ± SD^b^	49.8 ± 30.3	41.9 ± 24.6	
Range^b^	13.1–209.2	8.5–163.0	
≤40	83	150	
>40	109	103	
AST (IU/L)			0.115^a^
Mean ± SD^b^	41.6 ± 25.2	38.2 ± 20.4	
Range^b^	8.0–263.0	15.0–168.0	
≤45	138	189	
>45	54	64	
ALB (g/L)			0.485^a^
Mean ± SD^b^	43.1 ± 3.6	42.8 ± 4.0	
Range^b^	29.3–52.5	27.8–51.6	
<35	3	8	
≥35	189	245	
TBIL (μmol/L)			0.035^a^
Mean ± SD^b^	15.9 ± 7.3	14.6 ± 5.6	
Range^b^	4.6–63.3	5.0–47.2	
≤20.5	156	217	
>20.5	36	36	
Prothrombin time (s)			0.215^a^
Mean ± SD^b^	12.7 ± 1.5	12.5 ± 1.3	
Range^b^	9.6–19.1	9.4–17.4	
≤13.5	139	207	
>13.5	53	46	
Recurrence			0.489
Yes	105	130	
No	87	123	
Death			0.043
Yes	51	90	
No	141	163	

*Antiviral group* resection plus postsurgical antiviral treatment group, *Non-antiviral group* resection alone group, *HBV* hepatitis B virus, *SD* standard deviation, *HBeAg* hepatitis B e antigen, *TNM* tumor-node-metastasis, *AFP* alpha-fetoprotein, *ALT* alanine aminotransferase, *AST* aspartate aminotransferase, *ALB* serum albumin, *TBIL* serum total bilirubin

^ a^ *t* test used

^b^ Except for these values, other values are presented as the number of patients and were compared by the χ^2^ test

### OS and DFS analysis for all patients

#### OS

**Fig. 3:**
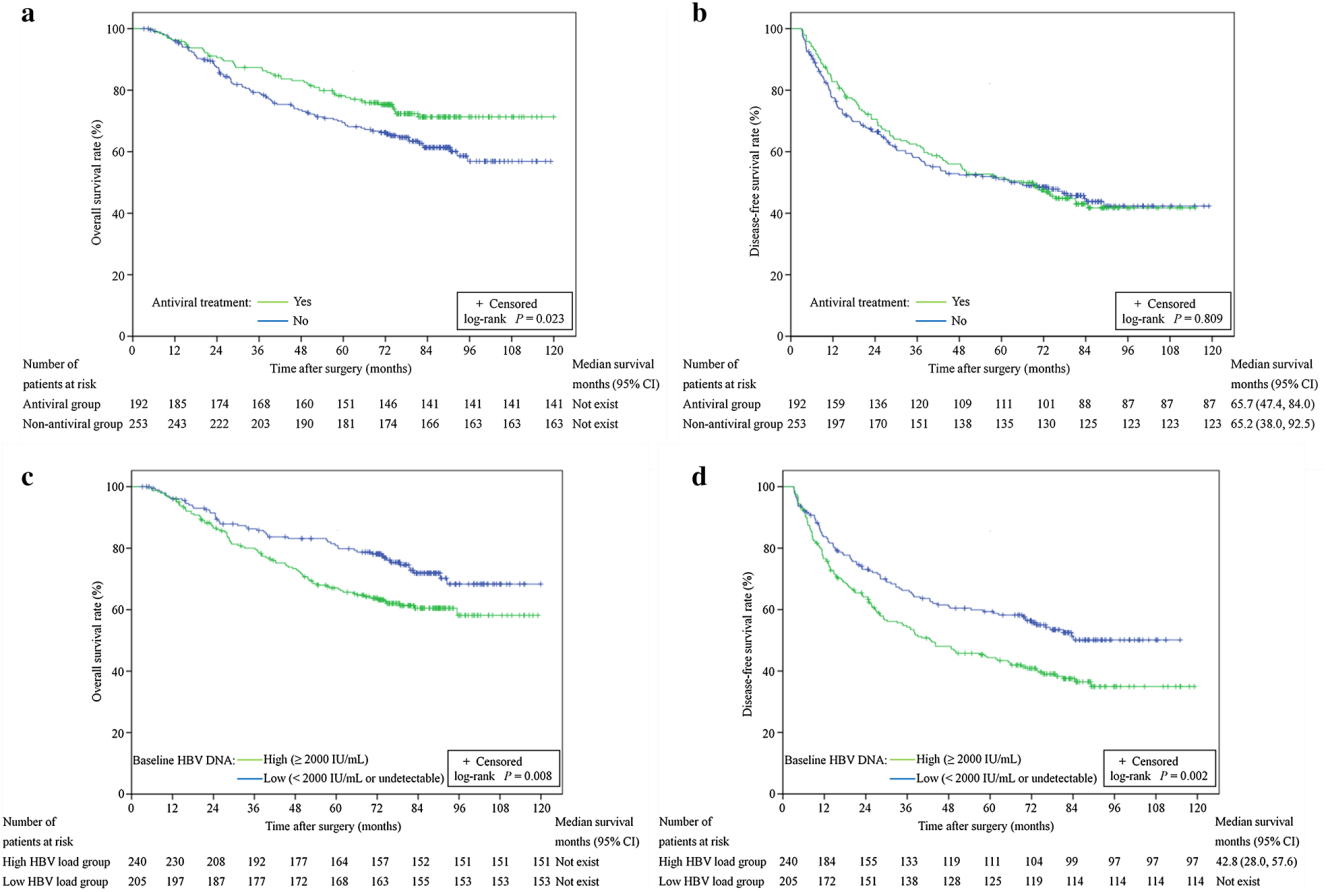
Kaplan–Meier survival curves of patients with various baseline HBV levels and use of nucleotide/nucleoside analogs (NAs). **a** OS rates between the antiviral and non-antiviral groups. **b** DFS rates between the antiviral and non-antiviral groups. **c** OS rates between patients with baseline serum HBV DNA levels of ≥2000 and <2000 IU/mL or undetectable. **d** DFS rates between patients with baseline serum HBV DNA levels of ≥2000 and <2000 IU/mL or undetectable

Patients in the antiviral group had higher OS rates than patients in the non-antiviral group (*P* = 0.023; Fig. 3a). The 1-, 3-, 5-, 7-, and 10-year OS rates in the antiviral group were 96.4 %, 86.9 %, 77.6 %, 71.3 %, and 71.3 %, respectively, whereas the corresponding rates in the non-antiviral group were 96.0 %, 78.8 %, 69.5 %, 61.4 %, and 56.9 %, respectively. Univariate analysis revealed that HBeAg, HBV DNA level, tumor size, tumor number, pathologic differentiation grade, microvascular thrombus, aspartate aminotransferase (AST) level, albumin (ALB) level, antiviral treatment, and recurrence were associated with OS (Table 2). On multivariate analysis, high baseline HBV DNA, multiple tumors, high pathologic grade, presence of microvascular thrombus, low ALB level, no antiviral treatment, and recurrence were independent risk factors that associated with short OS (Table 2).

**Table 2:** Relationship between clinical characteristics and overall survival (OS)/disease-free survival (DFS), as determined by univariate and multivariate Cox regression analysis

Variable	OS				DFS			
	Univariate analysis		Multivariate analysis		Univariate analysis		Multivariate analysis	
	HR	*P* value	HR (95 % CI)	*P* value	HR	*P* value	HR (95 % CI)	*P* value
Age (≥50 vs. <50 years)	1.320	0.101			1.116	0.401		
Gender (female vs. male)	0.951	0.845			0.921	0.684		
HBeAg (positive vs. negative)	1.582	0.020	Not significant		1.403	0.034	Not significant	
HBV DNA (≥2000 vs. <2000 IU/mL)	1.580	0.009	1.615 (1.126–2.317)	0.009	1.511	0.002	1.496 (1.150–1.944)	0.003
Tumor size (≥5 vs. <5 cm)	1.745	0.001	Not significant		1.687	<0.001	1.868 (1.432–2.435)	<0.001
Tumor number (multiple vs. single)	2.347	<0.001	1.930 (1.196–3.113)	0.007	1.961	0.001	2.437 (1.607–3.696)	<0.001
Pathologic grade (IV/III/II/I)	1.413	0.014	1.412 (1.064–1.875)	0.017	1.172	0.145		
Microvascular thrombus (yes vs. no)	2.076	0.002	2.339 (1.456–3.759)	<0.001	1.315	0.188		
Tumor capsule (without/incomplete/complete)	1.123	0.244			0.950	0.520		
AFP (>25 vs. ≤25 ng/mL)	1.181	0.335			1.079	0.568		
ALT (>40 vs. ≤40 IU/L)	1.249	0.188			1.212	0.141		
AST (>45 vs. ≤45 IU/L)	1.510	0.023	Not significant		1.597	0.001	Not significant	
ALB (≥35 vs. <35 g/L)	0.358	0.008	0.383 (0.176–0.833)	0.015	0.661	0.250		
TBIL (>20.5 vs. ≤20.5 μmol/L)	1.407	0.097	Not significant		1.227	0.222		
Prothrombin time (>13.5 vs. ≤13.5 s)	1.051	0.799			1.011	0.942		
Antiviral treatment (yes vs. no)	0.672	0.024	0.516 (0.359–0.743)	<0.001	0.969	0.809		
Recurrence (yes vs. no)	7.785	<0.001	7.208 (4.418–11.760)	<0.001				

*CI* confidence interval, *HR* hazard ratio, *Antiviral group* resection plus postsurgical antiviral treatment group, *Non-antiviral group* resection alone group, *HBV* hepatitis B virus, *SD* standard deviation, *HBeAg* hepatitis B e antigen, *TNM* tumor-node-metastasis, *AFP* alpha-fetoprotein, *ALT* alanine aminotransferase, *AST* aspartate aminotransferase, *ALB* serum albumin, *TBIL* serum total bilirubin

#### DFS

The 1-, 3-, 5-, 7-, and 10-year DFS rates in the antiviral group were 82.8 %, 61.9 %, 51.5 %, 41.8 %, and 41.8 %, respectively, whereas the corresponding rates in the non-antiviral group were 77.6 %, 57.7 %, 51.0 %, 43.8 %, and 42.3 %, respectively. No significant difference in DFS rates were observed between these two groups (*P* = 0.809; Fig. 3b). Univariate analysis showed that HBeAg, HBV DNA level, tumor size, tumor number, and AST level were associated with DFS (Table 2). On multivariate analysis, high baseline HBV DNA, tumor size larger than 5 cm, and multiple tumors were independent risk factors that associated with short DFS.

#### Baseline HBV DNA levels as a risk factor for both OS and DFS

Kaplan–Meier analysis revealed that the OS rates in patients with baseline HBV DNA <2000 IU/mL or undetectable were significantly higher than those in patients with HBV DNA levels ≥2000 IU/ml (*P* = 0.008; Fig. 3c). A similar result was found in the analysis of DFS rates (*P* = 0.002; Fig. 3d). Univariate and multivariate analyses demonstrated that high baseline HBV DNA was a risk factor for short DFS and OS (Table 2).

### Analysis based on the stratification of baseline HBV DNA and antiviral therapy

We stratified all patients by the levels of baseline HBV DNA between the antiviral and non-antiviral groups and evaluated the association of antiviral treatment with long-term prognosis in each stratum. Table 3 summarizes the characteristics of these four subgroups.

**Table 3:** Comparison of clinicopathologic characteristics among the subgroups

Variable	Subgroup 1 (*n* = 141)	Subgroup 2 (*n* = 51)	Subgroup 3 (*n* = 99)	Subgroup 4 (*n* = 154)	*P*_1_	*P*_2_	*P*_3_	*P*_4_
Age (years)					0.290^a^	0.042^a^	0.108^a^	0.526^a^
Mean ± SD^b^	48.4 ± 10.6	46.6 ± 9.6	50.7 ± 11.9	47.6 ± 11.7				
<50	78	33	41	87				
≥50	63	18	58	67				
Gender					0.216	0.527	0.988	0.092
Males	124	48	87	131				
Females	17	3	12	23				
HBeAg					0.011	<0.001	0.441	0.360
Positive	42	6	25	10				
Negative	99	45	74	144				
Tumor size (cm)					0.233^a^	0.922^a^	0.158^a^	0.020^a^
Mean ± SD^b^	4.8 ± 3.1	4.2 ± 2.8	5.4 ± 3.1	5.5 ± 3.3				
<5	86	36	45	76				
≥5	55	15	54	78				
Tumor number					0.460	0.194	0.278	0.360
Single	131	45	88	144				
Multiple	10	6	11	10				
Pathologic grade					0.561	0.375	0.498	0.444
I	10	2	12	9				
II	85	36	56	94				
III	45	13	30	50				
IV	1	0	1	1				
Microvascular thrombus					0.756	0.210	0.430	0.273
Yes	19	6	10	9				
No	122	45	89	145				
Tumor capsule (cases)					0.083	0.729	0.056	0.648
Complete	64	23	43	69				
Incomplete	49	11	24	42				
Without	28	17	32	43				
Liver cirrhosis					0.017	0.738	0.852	0.012
Yes	101	45	72	109				
No	40	6	27	45				
AFP (ng/mL)					0.216	0.514	0.897	0.523
≤25	61	17	42	59				
>25	80	34	57	95				
ALT (IU/L)					0.052^a^	<0.001^a^	0.485^a^	0.220^a^
Mean ± SD^b^	52.4 ± 29.3	42.8 ± 32.1	49.8 ± 26.0	36.8 ± 22.3				
≤40	53	30	46	104				
>40	88	21	53	50				
AST (IU/L)					0.009^a^	0.008^a^	0.508^a^	0.596^a^
Mean ± SD^b^	44.4 ± 27.1	33.7 ± 16.8	42.4 ± 17.6	35.5 ± 21.6				
≤45	96	42	62	127				
>45	45	9	37	27				
ALB (g/L)					0.028^a^	0.092^a^	0.372^a^	0.168^a^
Mean ± SD^b^	42.8 ± 3.5	44.1 ± 3.7	42.3 ± 4.0	43.2 ± 3.9				
<35	2	1	95	150				
≥35	139	50	4	4				
TBIL (μmol/L)					0.931^a^	0.878^a^	0.114^a^	0.193^a^
Mean ± SD^b^	16.0 ± 7.8	15.9 ± 5.8	14.5 ± 5.4	14.6 ± 5.8				
≤20.5	116	40	84	133				
>20.5	25	11	15	21				
Prothrombin time (s)					0.929^a^	0.182^a^	0.824^a^	0.345^a^
Mean ± SD^b^	12.7 ± 1.5	12.7 ± 1.6	12.6 ± 1.3	12.4 ± 1.2				
≤13.5	101	38	76	131				
>13.5	40	13	23	23				
Recurrence					0.535	0.001	0.180	0.312
Yes	79	26	64	66				
No	62	25	35	88				
Death					0.189	0.001	0.731	0.276
Yes	41	10	48	42				
No	100	41	51	112				

Definition of the four subgroups: (1) subgroup 1, antiviral therapy and baseline HBV DNA ≥ 2000 IU/mL (*n* = 141); (2) subgroup 2, antiviral therapy and baseline HBV DNA <2000 IU/mL or undetectable (*n* = 51); (3) subgroup 3, non-antiviral therapy and baseline HBV DNA ≥2000 IU/mL (*n* = 99); and (4) subgroup 4, non-antiviral therapy and baseline HBV DNA <2000 IU/mL or undetectable (*n* = 154)

*P*_*1*_ *P* value calculated by comparing subgroup 1 and subgroup 2, *P*_*2*_ *P* value calculated by comparing subgroup 3 and subgroup 4, *P*_*3*_ *P* value calculated by comparing subgroup 1 and subgroup 3, *P*_*4*_ *P* value calculated by comparing subgroup 2 and subgroup 4, *Antiviral group* resection plus postsurgical antiviral treatment group, *Non-antiviral group* resection alone group, *HBV* hepatitis B virus, *SD* standard deviation, *HBeAg* hepatitis B e antigen, *TNM* tumor-node-metastasis, *AFP* alpha-fetoprotein, *ALT* alanine aminotransferase, *AST* aspartate aminotransferase, *ALB* serum albumin, *TBIL* serum total bilirubin

^a^ *t* test used

^b^ Except for these values, other values are presented as the number of patients and were compared by the χ^2^ test

#### The association between baseline HBV DNA and the prognosis of the non-antiviral group (n = 253)

**Fig. 4:**
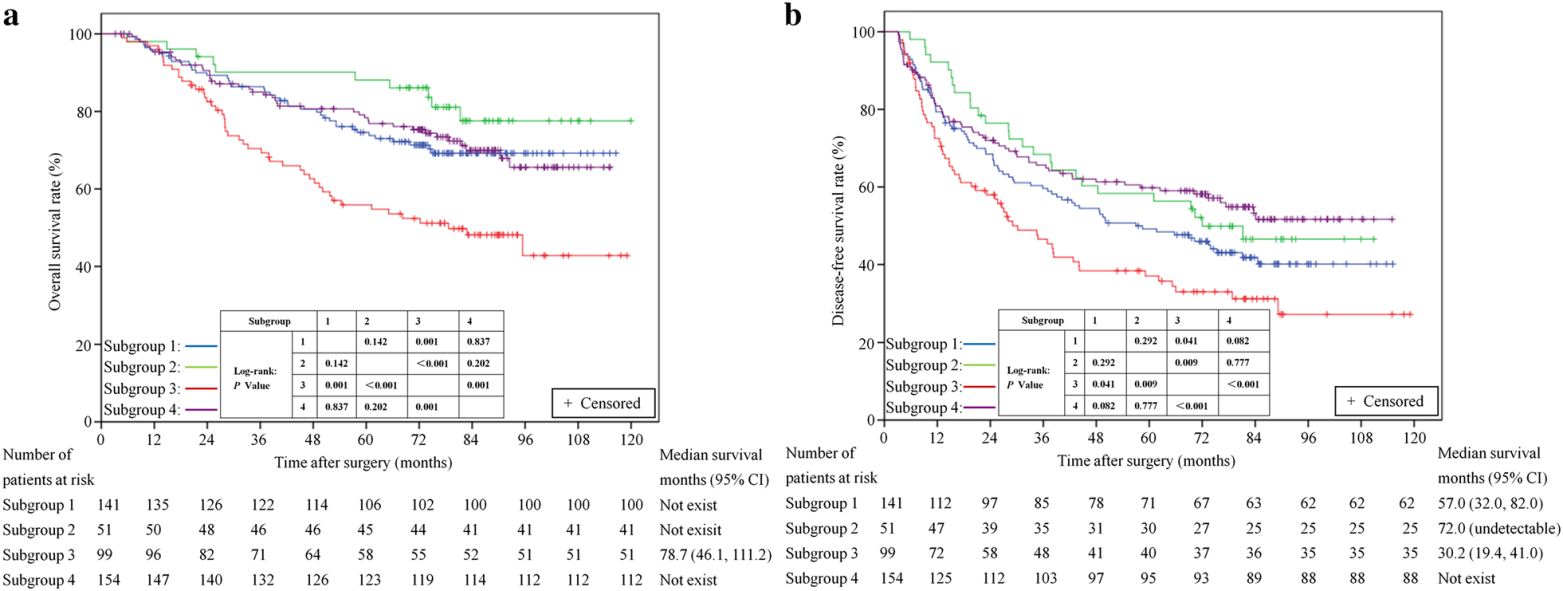
Kaplan–Meier survival curves of patients in antiviral and non-antiviral subgroups after stratification with baseline HBV DNA loads. **a** OS rates among the four subgroups of patients (log-rank test: subgroup 3 vs. subgroup 1, *P* = 0.001; subgroup 3 vs. subgroup 2, *P* < 0.001; subgroup 3 vs. subgroup 4, *P* = 0.001; subgroup 1 vs. subgroup 2, *P* = 0.142; subgroup 1 vs. subgroup 4, *P* = 0.837; subgroup 2 vs. subgroup 4, *P* = 0.202). **b** DFS rates among the four subgroups of patients (subgroup 3 vs. subgroup 1, *P* = 0.041; subgroup 3 vs. subgroup 2, *P* = 0.009; subgroup 3 vs. subgroup 4, *P* = 0.000; subgroup 1 vs. subgroup 2, *P* = 0.292; subgroup 1 vs. subgroup 4, *P* = 0.082; subgroup 2 vs. subgroup 4, *P* = 0.777). [Note: (1) Subgroup 1, antiviral treatment with baseline hepatitis B virus (HBV) DNA ≥2000 IU/mL (*n* = 141); (2) Subgroup 2, antiviral treatment with baseline HBV DNA <2000 IU/mL or undetectable (*n* = 51); (3) Subgroup 3, no antiviral treatment with baseline HBV DNA ≥2000 IU/mL (*n* = 99); and (4) Subgroup 4, no antiviral treatment with baseline HBV DNA <2000 IU/mL or undetectable (*n* = 154).]

For the 253 patients in the non-antiviral group (subgroups 3 and 4), Kaplan–Meier analysis showed that higher baseline HBV DNA levels were associated with lower DFS and OS rates (Fig. 4a for OS analysis, *P* = 0.001; Fig. 4b for DFS analysis, *P* < 0.001). Univariate and multivariate Cox models showed that high baseline HBV DNA along with some tumor characteristics (multiple tumors, tumor size larger than 5 cm, and presence of microvascular thrombus) were risk factors for short DFS and OS in the non-antiviral group (Table 4).

**Table 4:** Relationship between clinical characteristics and OS/DFS in patients without antiviral treatment (*n* = 253), as determined by univariate and multivariate Cox regression analysis

Variable	OS				DFS			
	Univariate analysis		Multivariate analysis		Univariate analysis		Multivariate analysis	
	HR	*P* value	HR (95 % CI)	*P* value	HR	*P* value	HR (95 % CI)	*P* value
Age (≥50 vs. <50 years)	1.341	0.169			0.933	0.693		
Gender (female vs. male)	0.891	0.710			0.827	0.465		
HBeAg (positive vs. negative)	1.681	0.054	Not significant		1.716	0.019	Not significant	
HBV DNA (≥2000 vs. <2000 IU/mL)	2.056	0.001	1.589 (1.042–2.422)	0.031	1.840	0.001	1.705 (1.204–2.415)	0.003
Tumor size (≥5 vs. <5 cm)	1.820	0.006	1.689 (1.087–2.624)	0.020	1.655	0.005	1.713 (1.195–2.455)	0.003
Tumor number (multiple vs. single)	2.393	0.004	2.259 (1.223–4.172)	0.009	2.227	0.004	2.571 (1.470–4.499)	0.001
Pathologic grades (IV/III/II/I)	1.357	0.075	Not significant		1.288	0.074	Not significant	
Microvascular thrombus (yes vs. no)	2.915	0.001	2.587 (1.345–4.978)	0.004	1.969	0.021	1.192 (1.066–3.429)	0.030
Tumor capsule (without/incomplete/complete)	1.028	0.825			0.880	0.226		
AFP (>25 vs. ≤25 ng/mL)	1.252	0.302			1.064	0.728		
ALT (>40 vs. ≤40 IU/L)	1.539	0.041	Not significant		1.375	0.072	Not significant	
AST (>45 vs. ≤45 IU/L)	1.803	0.009	Not significant		1.687	0.006	Not significant	
ALB (≥35 vs. <35 g/L)	0.423	0.061	Not significant		0.643	0.291		
TBIL (>20.5 vs. ≤20.5 μmol/L)	0.919	0.780			1.126	0.618		
Prothrombin time (>13.5 vs. ≤13.5 s)	1.146	0.593			1.015	0.945		
Recurrence (yes vs. no)	6.671	<0.001	5.442 (3.091–9.582)	<0.001				

*OS* overall survival, *DFS* disease-free survival, *CI* confidence interval, *HR* hazard ratio, *Antiviral group* resection plus postsurgical antiviral treatment group, *Non-antiviral group* resection alone group, *HBV* hepatitis B virus, *SD* standard deviation, *HBeAg* hepatitis B e antigen, *TNM* tumor-node-metastasis, *AFP* alpha-fetoprotein, *ALT* alanine aminotransferase, *AST* aspartate aminotransferase, *ALB* serum albumin, *TBIL* serum total bilirubin

#### The effect of antiviral treatment on the prognosis of patients with high baseline HBV DNA (≥2000 IU/mL)

In the antiviral group with high baseline HBV DNA, the OS rate was significantly higher than that of the non-antiviral group (Fig. 4a, subgroup 1 vs. subgroup 3, *P* = 0.001). Both univariate and multivariate analyses identified antiviral treatment as a risk factor linked to OS (Table 5). As to the analysis of DFS, antiviral treatment conferred a significantly higher DFS rate (Fig. 4b, subgroup 1 vs. subgroup 3, *P* = 0.041). On univariate analysis, tumor size, tumor number, AST level, and antiviral treatment were significantly associated with DFS. On multivariate analysis, tumor size, tumor number, and AST level were independent risk factors linked to DFS, whereas antiviral treatment was not an independent protective factor of DFS in the model (Table 5).

**Table 5:** Relationship between clinical characteristics and OS/DFS in patients with high HBV DNA level (≥2000 IU/mL), as determined by univariate and multivariate Cox regression analysis

Variable	OS				DFS			
	Univariate analysis		Multivariate analysis		Univariate analysis		Multivariate analysis	
	HR	*P* value	HR (95 % CI)	*P* value	HR	*P* value	HR (95 % CI)	*P* value
Age (≥50 vs. <50 years)	1.329	0.186			1.384	0.055	Not significant	
Gender (female vs. male)	1.087	0.795			0.914	0.737		
HBeAg (positive vs. negative)	1.407	0.132			1.216	0.290		
Tumor size (≥5 vs. <5 cm)	1.618	0.024	Not significant		1.618	0.004	1.650 (1.162–2.342)	0.005
Tumor number (multiple vs. single)	2.175	0.010	Not significant		1.796	0.028	2.104 (1.218–3.635)	0.008
Pathologic grades (IV/III/II/I)	1.365	0.070	1.613 (1.129–2.305)	0.009	1.100	0.476		
Microvascular thrombus (yes vs. no)	2.038	0.010	2.688 (1.534–4.710)	0.001	1.128	0.640		
Tumor capsule (without/incomplete/complete)	1.201	0.148			1.030	0.771		
AFP (>25 vs. ≤25 ng/mL)	1.291	0.240			1.030	0.863		
ALT (>40 vs. ≤40 IU/L)	1.064	0.774			1.088	0.621		
AST (>45 vs. ≤45 IU/L)	1.657	0.018	Not significant		1.688	0.002	1.486 (1.057–2.090)	0.023
ALB (≥35 vs. <35 g/L)	0.294	0.008	Not significant		0.633	0.316		
TBIL (>20.5 vs. ≤20.5 μmol/L)	1.566	0.077	Not significant		1.098	0.672		
Prothrombin time (>13.5 vs. ≤13.5 s)	0.867	0.557			0.814	0.283		
Antiviral treatment (yes vs. no)	0.499	0.001	0.475 (0.309–0.729)	0.001	0.710	0.042	Not significant	0.120
Recurrence (yes vs. no)	9.514	<0.001	9.954 (4.781–20.725)	<0.001				

*OS* overall survival, *DFS* disease-free survival, *CI* confidence interval, *HR* hazard ratio, *Antiviral group* resection plus postsurgical antiviral treatment group, *Non-antiviral group* resection alone group, *HBV* hepatitis B virus, *SD* standard deviation, *HBeAg* hepatitis B e antigen, *TNM* tumor-node-metastasis, *AFP* alpha-fetoprotein, *ALT* alanine aminotransferase, *AST* aspartate aminotransferase, *ALB* serum albumin, *TBIL* serum total bilirubin

#### The effect of antiviral treatment on the prognosis of patients with low (<2000 IU/mL) or undetectable baseline HBV DNA

Kaplan–Meier analysis showed no significant difference in OS rates between antiviral and non-antiviral treatment groups in patients with low or even undetectable baseline HBV DNA (Fig. 4a, subgroup 2 vs. subgroup 4, *P* = 0.202). Univariate analysis identified the following factors as significantly associated with OS: tumor size, tumor number, and recurrence. On multivariate analysis, only recurrence was an independent risk factor linked to OS (Table 6). As to the analysis of DFS, no significant difference was observed in DFS rates between the antiviral and non-antiviral groups in patients with low or even undetectable baseline HBV DNA (Fig. 4b, subgroup 2 vs. subgroup 4, *P* = 0.777). Both univariate and multivariate analyses showed that tumor number and tumor size were prognostic factors related to DFS (Table 6). Antiviral treatment was not a risk factor related to OS or DFS in the analysis.

**Table 6:** Relationship between clinical characteristics and OS/DFS in patients with undetectable or low baseline HBV DNA levels (<2000 IU/mL), as determined by univariate and multivariate Cox regression analysis

Variable	OS				DFS			
	Univariate analysis		Multivariate analysis		Univariate analysis		Multivariate analysis	
	HR	*P* value	HR (95 % CI)	*P* value	HR	*P* value	HR (95 % CI)	*P* value
Age (≥50 vs. <50 years)	1.173	0.566			0.750	0.183		
Gender (female vs. male)	0.819	0.645			0.963	0.902		
HBeAg (positive vs. negative)	1.321	0.554			1.407	0.330		
Tumor size (≥5 or <5 cm)	2.013	0.013	Not significant		1.772	0.006	1.997 (1.295–3.044)	0.002
Tumor number (multiple vs. single)	2.612	0.013	Not significant		2.194	0.015	2.761 (1.440–5.296)	0.002
Pathologic grades (IV/III/II/I)	1.535	0.073	Not significant		1.346	0.100	Not significant	
Microvascular thrombus (yes vs. no)	1.902	0.140			1.561	0.205		
Tumor capsule (without/incomplete/complete)	1.046	0.784			0.873	0.284		
AFP (>25 vs. ≤25 ng/mL)	1.084	0.777			1.230	0.345		
ALT (>40 vs. ≤40 IU/L)	1.219	0.487			1.115	0.618		
AST (>45 vs. ≤45 IU/L)	0.820	0.626			1.107	0.712		
ALB (≥35 vs. <35 g/L)	0.490	0.324			0.712	0.562		
TBIL (>20.5 vs. ≤20.5 μmol/L)	1.167	0.661			1.439	0.158		
Prothrombin time (>13.5 vs. ≤13.5 s)	1.296	0.432			1.272	0.342		
Antiviral treatment (yes vs. no)	0.641	0.206			1.068	0.777		
Recurrence (yes vs. no)	5.859	<0.001	5.859 (3.007–11.416)	<0.001				

*OS* overall survival, *DFS* disease-free survival, *CI* confidence interval, *HR* hazard ratio, *Antiviral group* resection plus postsurgical antiviral treatment group, *Non-antiviral group* resection alone group, *HBV* hepatitis B virus, *SD* standard deviation, *HBeAg* hepatitis B e antigen, *TNM* tumor-node-metastasis, *AFP* alpha-fetoprotein, *ALT* alanine aminotransferase, *AST* aspartate aminotransferase, *ALB* serum albumin, *TBIL* serum total bilirubin

#### Patients with high HBV DNA load and no antiviral treatment had the worst prognosis

Kaplan–Meier analysis showed that subgroup 3 had a significantly lower OS rate compared with the other three subgroups (subgroup 3 vs. subgroup 1, *P* = 0.001; subgroup 3 vs. subgroup 2, *P* = 0.000; subgroup 3 vs. subgroup 4, *P* = 0.001). Among subgroups 1, 2, and 4, the differences were not significant (Fig. 4a).

As for DFS, similarly, subgroup 3 had a significantly lower DFS rate compared with the other three subgroups (subgroup 3 vs. subgroup 1, *P* = 0.041; subgroup 3 vs. subgroup 2, *P* = 0.009; subgroup 3 vs. subgroup 4, *P* = 0.000). Subgroup 4 appeared to have a higher DFS rate than subgroup 1, but the difference was not significant (*P* = 0.082). As to the comparisons between subgroups 2 and 4 and between subgroups 1 and 2, the differences were not significant (Fig. 4b).

## Discussion

In the present study, we found that baseline HBV DNA level was a significant prognostic factor that influenced both OS and DFS of early-stage HCC patients undergoing curative resection, which was consistent with the findings of most previous studies [24–27]. The precise mechanism for recurrent carcinogenesis in patients with high HBV DNA levels remains unclear. It is possible that active viral replication may contribute to hepatocarcinogenesis by direct and indirect pathways [28]. Clearly, recurrence tends to shorten OS of cancer patients. One study showed that sustained low HBV load below 2000 IU/mL was a strong protective factor for long-term DFS and OS after curative resection in HBV-related HCC [23]. In addition, the effect of NAs differs in patients with different tumor stages [16, 29]. Therefore, HBV DNA level, tumor stage, and liver function should be considered before administration of NAs [18].

In the present study, no significant differences were observed in DFS rates between the antiviral and non-antiviral groups, whereas the OS rates were higher in the antiviral group than in the non-antiviral group. Treatment was an independent prognostic factor related to OS, which is consistent with the results of a prospective-retrospective study carried out in Hong Kong [30]. However, other studies reported that antiviral therapy reduced the risk of recurrence of HCC after curative therapy [14, 15]. The discrepancy in the results regarding tumor recurrence could be due to different study populations (including tumor stage) and study designs. As to the effect of antiviral therapy on OS, there are several possible explanations. First, antiviral treatment could reduce the risk of HBV reactivation, and subsequent liver dysfunction after surgery, which would reduce perioperative liver-related mortality [20, 31]. Second, better liver function preservation at the time of recurrence could translate into a high proportion of patients receiving more aggressive curative or palliative treatment at the time of recurrence to extend OS [30, 32]. Third, although not well defined, antiviral therapy can decrease the hepatitis activity caused by HBV, leading to a reduced risk of late-phase recurrence of HCC, which can therefore increase the OS rate [33].

In the stratification analyses, antiviral treatment was associated with better prognosis (both OS rate and DFS rate) in patients with high baseline HBV DNA levels. However, in patients with low or undetectable baseline HBV DNA levels, no significant difference was found in either DFS rate or OS rate between the antiviral and non-antiviral groups. In this population, univariate and multivariate analysis did not identify antiviral treatment as a risk factor for DFS or OS. A previous study demonstrated that patients with persistently low HBV DNA levels had better survival results compared with those with high or fluctuating HBV DNA levels [23]. Although the stratification in our study was based on the evaluation of baseline HBV DNA at resection, most (135/154, 87.6 %) of the HBV DNA levels in subgroup 4 remained stable during the follow-up period (data not shown). In 33.1 % (51/154) of the patients, serum HBV DNA was even undetectable. The results indicated that HCC patients with low or undetectable HBV DNA load may not significantly benefit from antiviral therapy as patients with high HBV DNA load did.

However, some weaknesses could be noted in our study. First, the proportion of para-tumorous liver cirrhosis was higher in subgroup 2 (Table 3), which may be explained by the fact that patients with cirrhosis were more likely to be treated with NAs. Second, the mean tumor size in subgroup 2 was smaller than that in subgroup 4, which might have positively affected the outcome of the antiviral group. Third, administration of NAs was not randomized, which may have caused selection bias. In addition, for the OS curves, a small gap was observed between these two subgroups (subgroups 2 and 4), although no significant difference existed. Therefore, the results should be interpreted very carefully.

So far, no consensus exists on the indications of antiviral therapy for HCC patients with low HBV DNA load. Many guidelines have recommended NAs for patients with chronic HBV infection, evidence of active viral replication (≥2000 IU/mL), and elevated levels of ALT [18]. It remains controversial whether HCC patients with low HBV replication should be given antiviral therapy. In a study aimed to analyze the different prognosis after hepatectomy of HBV-related HCC with or without cirrhosis, the results revealed that antiviral treatment was an independent predictor for prolonged OS in HCC patients with cirrhosis [34]. However, in patients without cirrhosis, antiviral therapy did not significantly associated with either prolonged OS or prolonged DFS after radical resection, which indicated that HCC patients with cirrhosis benefit more from antiviral therapy than do those without cirrhosis. In a two-stage longitudinal clinical study, the investigators found that antiviral therapy reduced HCC recurrence in patients with a low HBV load [13]. However, that study included patients with early Barcelona clinical liver cancer (BCLC) stage (0 to A) or intermediate stage (B), and the percentage of BCLC B stage in the non-antiviral group was nearly twice that of the antiviral group. Furthermore, several imbalances were observed between the antiviral and non-antiviral groups, including tumor encapsulation, tumor differentiation, and AFP [13]. Thus, the findings are not conclusive. Most recently, several studies showed that high levels of HBsAg were associated with short survival and early recurrence after surgery for HCC patients with low HBV viral loads [35–37], which may provide a clue for selecting suitable candidates for antiviral therapy from HCC patients with low viral load. However, further investigation is needed to determine the magnitude of benefit and to clarify whether NAs should be administered to all or only a subset of patients (such as patients with cirrhosis or high levels of HBsAg) after curative treatment for HBV-related HCC [33].

Our study also showed that DFS rate tended to be higher in the non-antiviral subgroup with low or undetectable HBV DNA level than in the antiviral subgroup with high HBV DNA levels, although the OS rate was similar. Cho et al. [38] recently found that patients with active chronic hepatitis B (CHB) who were receiving long-term NA treatment had a higher incidence of HCC compared with patients with inactive CHB. One possible explanation is that the inactive CHB group may have had more intact immune response to HBV and, therefore, the HBV infection may have entered the inactive stage early in the patient’s life. However, in patients with high viral replication and active hepatitis, HBV DNA integration into hepatocytes that resulted in genomic alterations and/or chromosomal instability may have already occurred before initiation of NA treatment. This may explain why, in our study, patients in subgroup 4 had a higher DFS rate than patients in subgroup 1 who were treated with NAs after surgery. However, a more detailed investigation is needed to confirm these results.

In the present retrospective study, we included only the patients with TNM stage I and II disease who had normal liver function before surgery (Child-Pugh grade A); we excluded those who experienced severe complications shortly after surgery. We evaluated only patients who were likely to receive curative treatment and tolerate it well. Therefore, the conclusions may not be generalizable to all HCC patients undergoing surgery. During the treatment period between January 2005 and December 2008, whether it was beneficial to give antiviral therapy to HBV-related HCC patients who underwent resection was controversial at our institution. At that time, the major liver society guidelines for CHB only recommended that patients who had persistently elevated ALT levels more than two times the upper limit of normal should be considered for antiviral treatment. Decisions on antiviral treatment were made on a case-by-case basis, which may have caused selection bias. Therefore, prospective large-scale trials, with hepatitis B and cirrhotic HCC patients at various stages, will be required in the future to clarify the effects of antiviral therapy on survival and HCC recurrence [39].

In conclusion, high baseline HBV DNA levels were associated with poor prognosis. For the patients with high HBV replication, antiviral treatment significantly improved patients’ long-term prognoses, including DFS and OS. However, for the patients with low or even undetectable HBV DNA levels, whether the use of NAs is beneficial remains unclear and requires further investigation.
